# Apoplexy

**Published:** 1848-01

**Authors:** W. W. Reid

**Affiliations:** Rochester, N. Y.


ART. II.—*Apoplexy.—Three cases; two fatal, one recovered.* By W. W. Reid, M. D., Rochester, N. Y.

Dr. Flint,—*Dear Sir*—The public prints had just announced the painful intelligence, that a distinguished clergyman of your city, had been stricken down by apoplectic paralysis, when our own community was thrown into consternation and mourning, by the sudden seizure and death of one of our most worthy citizens by the same disease.

The frequent notices of death by apoplexy, in the newspapers, would seem to indicate that this appalling and fatal malady is becoming quite common. For, as generally speaking, persons only of some distinction are thus noticed, it may be presumed that those of less note, are not published. At least three cases have occurred in our city within the last sixteen weeks. One was an old gentleman 77 years of age—formerly a practising physician. He died instantly, just after he had seated himself at the tea-table, and had commenced eating—without a groan, or struggle, and without the least warning. No examination of the body was made. The two other cases fell under my own immediate care; of these I propose to speak more particularly, not because they present anything novel, or unknown to the profession, but because of the opposite conditions attending them.

*Case first.*—E. S., a butcher, 25 years of age, of medium size, sanguineous temperament, with strong digestive and assimilative powers; for 4 or 5 months had had a roaring and hissing noise in the ears, with deafness—also pain in the back and left side of the head; stricture through the temples, and pressure on the eyeballs. On the 24th of July last, after working hard all the forenoon, and eating a hearty dinner, at 3 o'clock, P. M., while at work in the market, was suddenly seized with dizziness, which lasted (as he afterwards said) one or two minutes, when he felt as if a ton weight was put upon his right side; he seized a post to hold himself up, and remembered nothing more. In ten minutes afterward I saw him; found him lying on the floor, unconscious, frothing at the mouth, countenance flushed, breathing irregular and laborious, pulse, slow, full and strong; muscles of the right side paralytic, those of the left, trembling and twitching. I immediately opened a vein; drew two pints of blood, when he opened his eyes; the spasms ceased, and he attempted to speak. I directed and assisted him to thrust the index finger of the left hand into his fauces, which he did, most perseveringly, till he threw up two-thirds of his dinner, consisting of meat, potatoes, carrots, bread, &c., nearly as when first eaten. An emetic of Ipecac. was immediately given, which with the aid of his finger, soon brought away the remainder of his meal. Subsequent treatment:—Drastic cathartics, one venesection, abstemious diet and rest. In five days the paralysis had all disappeared; in two weeks he was about his business; but two weeks subsequently, the noise in his ears and pain in his head returned, to subdue which, he has since been repeatedly bled, and cupped on the nape of the neck; taken active purges, restricted to a spare vegetable diet, and for the purpose of controlling his digestive and assimilative powers, taken in solution 1-8 gr. Tart. Ant. one hour before each meal—is now (Dec. 1.) quite free from all untoward symptoms.

*Case second.*—D. S., aet. 53., of highly bilious temperament, dark, sallow skin, medium size, sedentary habits, and for many years a dyspeptic; of very temperate and uniform habits, not accustomed to labor, and took very little exercise by way of recreation. On the morning of the 26 Nov. took a spare breakfast, assisted in lifting and adjusting some furniture, went to the bank of which he was an officer, and performed his usual business. About 10 minutes past 12 o'clock, M. while seated at a table, transacting some business with two gentlemen, he suddenly laid off his spectacles, partly arose from his chair, and requested the gentlemen to assist him to a sofa—at the same moment seizing his left hand with his right and rubbing it, which, they (the gentlemen) observed was cramped. They immediately took hold of him to aid him, when he began to tremble, and sink towards the floor, which they prevented, and laid him on the sofa. While being placed on the sofa, he said "send for Dr. Moore" (his family physician)—"send for my wife"—immediately became unconscious, and never spoke again. Dr. M. being absent, I saw him within 10 minutes after the attack; found him unconscious, frothing at the mouth, respiration stertorious, countenance natural, opened and closed his eyes twice, muscles of the left side paralytic, those of the right trembling, and twitching, pulse about 35 per minute. The action of the heart, tumultuous and violent. Opened a vein and drew three pints of blood, which was very dark and ran very slowly. Administered a stimulating enema, applied strong sinnpisms extensively to all the extremities, and hot bricks to the feet, but no amendment of the symptoms. After consultation with Drs. Ely and Tobey, it was agreed to draw more blood, as the pulse was still very full and resisting, and about 80 to 90 pr. mt. Accordingly 2 pints more were drawn before the pulse became softer and more frequent—about 100. The respiration was more free between the paroxysms of choaking and struggling, when the respiration would become nearly suspended and the lips and face became quite livid. Then paroxysms would recur about every 25 minutes, and grew more violent, notwithstanding the intervals seemed more free. In one of these paroxysms, 3 hours from the attack, he expired.

Autopsy, 20 hours after death.—Lungs; a few tubercles in a state of softening, in the superior lobe of the right side; a few adhesions posterially. Heart; left ventricle considerably hypertrophied, valves sound, Stomach; perfectly empty, but dotted throughout in the mucus membrane with deep red spots, from one to three lines in diameter. Bladder; distended, and the mucus membrane of the fundus, including about one fourth of the whole surface, of a deep red color, like the spots in the stomach. Brain; in situ, presented a dark conjested appearance—two or three inches on each hemisphere on the superior, posterior parts of the crebrum, was of a lighter color, and seemed more diffused, as if bloody serum was effused under the pia mater. On removal from the skull, some bloody serum was found at the base, the serum of the ventricles was also bloody, though not more abundant than natural. But in the substance of the cerebellum, just above and behind the medulla oblongata, was found a dark coagulum to the amount of half a fluid ounce; there were also two or three small ones of the size of a pea, in the neighborhood of the large coagulum.

I should have remarked, that half an hour after the attack, the right side of the body became as completely paralysed as the left. The situation of the coagulum; the direct pressure that it must have produced on the medulla oblongata, fully accounts for all the symptoms, especially the general paralysis, and the consequent difficulty of respiration; the frequent and almost entire suspension of the latter before its final cessation, which was the immediate cause of the speedily fatal termination of the case.

The contrariety of these two cases may be summed up thus:—The first, young 25; the second, 53 years of age. The first, of sanguineous temperament; the second, bilious; the one, of active and laborious habits; the other, sedentary; the one, possessing strong digestive and assimilative powers; the other, feeble, and dyspeptic; the former, had long had warning, by unmistakable symptoms; the latter, had none; the first, had worked hard and eaten heartily, and of course the stomach was full at the moment of attack; the second, had done no physical labor of any consequence, had eated sparingly six hours before, and of course the stomach was empty; the first, was paralytic on the right side; the second, on the left; the one recóvered; the other died.

ROCHESTER, Dec., 1847.

